# Impact of Enzymatic Degradation Treatment on Physicochemical Properties, Antioxidant Capacity, and Prebiotic Activity of Lilium Polysaccharides

**DOI:** 10.3390/foods14020246

**Published:** 2025-01-14

**Authors:** Kaitao Peng, Yujie Zhang, Qi Zhang, Yunpu Wang, Yuhuan Liu, Xian Cui

**Affiliations:** 1 State Key Laboratory of Food Science and Resources, Engineering Research Center for Biomass Conversion, Ministry of Education, Nanchang University, Nanchang 330047, China; pkt1811826@163.com (K.P.); jialishena@163.com (Y.Z.); zhangqi093115@ncu.edu.cn (Q.Z.); wangyunpu@ncu.edu.cn (Y.W.); 2 Chongqing Research Institute, Nanchang University, Chongqing 402660, China

**Keywords:** *Lilium* polysaccharide, fermentation characteristics, gut microbiota, metabolites

## Abstract

In order to overcome the bioavailability limitation of *Lilium* polysaccharide (LPS) caused by its high molecular weight and complex structure, two low-molecular-weight degraded polysaccharides, namely G-LPS(8) and G-LPS(16), were prepared through enzymatic degradation. The molecular weight of LPS was significantly reduced by enzymolysis, leading to increased exposure of internal functional groups and altering the molar ratio of its constituent monosaccharides. The results of antioxidant experiments showed that enzymatic hydrolysis had the potential to enhance the antioxidant performance of LPS. *In vitro* fermentation experiments revealed that LPS and its derivatives exerted different prebiotic effects on intestinal microbial communities. Specifically, LPS mainly inhibited the growth of harmful bacteria such as Fusobacterium, while G-LPS(8) and G-LPS(16) tended to promote the growth of beneficial bacteria like *Megamonas*, *Bacteroides*, and *Parabacteroides*. Metabolomic analysis revealed that LPSs with varying molecular weights exerted comparable promoting effects on multiple amino acid and carbohydrate metabolic pathways. Importantly, with the reduction in molecular weight, G-LPS(16) also particularly stimulated sphingolipid metabolism, nucleotide metabolism, as well as ascorbic acid and uronic acid metabolism, leading to the significant increase in specific metabolites such as sphingosine. Therefore, this study suggests that properly degraded LPS components have greater potential as a prebiotic for improving gut health.

## 1. Introduction

*Lilium brownii var. viridulum*, a common variety of Chinese medicinal and dietary herbs, is prepared from its dried bulb scales [1]. The primary bioactive constituent of lily bulbs is polysaccharide [2]. Clinical research has demonstrated that *Lilium* polysaccharide (LPS) possesses significant pharmacological properties, including hypoglycemic effects [3], antioxidant activity [4], immunomodulatory functions [5], and regulation of intestinal microecology [6]. However, natural polysaccharides often exhibit high molecular weight (Mw), elevated viscosity, and intricate structures, which substantially impede their absorption and utilization within the human body and hinder the broad application and development of active polysaccharides [7]. Consequently, identifying an environmentally friendly and efficient degradation technique to decrease Mw and viscosity, while enhancing functionality, could provide a theoretical basis for the progress of functional foods. Literature indicates that the principal methods for polysaccharide degradation encompass chemical, physical, and biological approaches [8]. Among these, enzymatic degradation, a subset of biological degradation, has emerged as the predominant technology due to its benefits, such as mild reaction conditions, precise product control, and high substrate specificity.

Recent research has shown that bioactive polysaccharides are not fully digested or absorbed in the human gastrointestinal system; instead, they serve as substrates for metabolism by intestinal flora, thereby exerting beneficial effects on the host [9,10]. Prior research has highlighted that the Mw of polysaccharides constitutes a critical factor influencing their colonic fermentation properties [11,12]. For instance, the investigations conducted by Sun et al. have revealed that fucoidans named Dfu2, characterized by a lower Mw, exhibit enhanced microbial availability in the intestine and a more pronounced regulation of the metabolite profile [13]. Nevertheless, to the best of our knowledge, there is a paucity of data concerning the structural alterations and fermentation behavior of LPS subjected to enzymatic degradation. Additionally, limited studies have explored the impact of enzymatically degraded derivatives of LPS on the intestinal microbiota and their metabolites.

In this study, two types of *Lilium* polysaccharides, G-LPS(8) and G-LPS(16), were prepared using enzymatic hydrolysis. The effects of enzymatic degradation on the physicochemical properties of these polysaccharides were systematically evaluated through measurements of sugar content, monosaccharide composition, and molecular weight. Additionally, the antioxidant activity of the *Lilium* polysaccharides before and after enzymatic treatment was assessed by evaluating their free radical scavenging capacity using DPPH and ABTS assays. Furthermore, this research explored the impact of enzymatic hydrolysis on probiotic activity by establishing an *in vitro* colon anaerobic fermentation model, with a focus on how enzymatic degradation influences the regulation of gut microbiota and the production of associated metabolites. The primary objective of this study was to elucidate the effects of enzymatic degradation on the structural characteristics, antioxidant activity, and prebiotic potential of *Lilium* polysaccharides, thereby providing valuable insights and a scientific foundation for their comprehensive utilization.

## 2. Materials and Methods

### 2.1. Materials and Reagents

*Lilium* polysaccharide (LPS) (average Mw = 67.3 kDa) was purchased from Shanghai yuanye Bio-Technology Co., Ltd (Shanghai, China), β-glucanase (5.0 × 10^4^ U/g) from Beijing Solarbio Science&Technology Co., Ltd. (Beijing, China), and the monosaccharide standard was purchased from Sigma-Aldrich Co., Ltd. (St. Louis, MO, USA). Trifluoroacetic acid (TFA) and methanol (chromatographic grade) were purchased from ANPEL Laboratory Technologies (Shanghai) Inc. (Shanghai, China). All other chemicals and reagents were of analytical grade unless specified and purchased from Shanghai Macklin Biochemical Technology Co., Ltd (Shanghai, China).

### 2.2. The Preparation of G-LPS(8) and G-LPS(16)

A precise amount of LPS powder was weighed and dissolved in deionized water at a solid-to-liquid ratio of 1:100. Subsequently, β-glucanase was added at amounts of 0.8 × 10^3^ U/g and 1.6 × 10^3^ U/g, respectively, and stirred at 50 °C for enzymatic hydrolysis for 6 h. After enzymolysis, the enzyme was inactivated by boiling for 10 min. Afterward, the mixture was subjected to centrifugation at 5000× *g* for 10 min, and the resulting supernatant was collected. The supernatant was then placed in a rotary evaporator (RE2000A, Shanghai Yarong Biochemical Instrument Co., Ltd., Shanghai, China) to concentrate it to the desired volume. The Sevag method was employed to remove the protein content [14]. Finally, it was dialyzed for 48 h with a 500 Da dialysis bag and then freeze-dried to produce enzymatically degraded LPS samples. According to the amount of β-glucanase added during the reaction of 0.8 × 10^3^ U/g and 1.6 × 10^3^ U/g, the degraded samples were, respectively, named G-LPS(8) and G-LPS(16).

### 2.3. Chemical Composition Determination

The total sugar content was determined by a phenol-sulfuric acid method [15]. The reducing sugar content was determined by a DNS method [16]. All samples were measured in parallel three times.

### 2.4. Structural Characterization

#### 2.4.1. Molecular Weight Determination

The relative molecular mass distribution of LPS, G-LPS(8), and G-LPS(16) was analyzed using high-performance gel permeation chromatography (HPGPC). The specific method is referred to the method described by Hou et al. and modified slightly [17]. Samples were prepared by dissolving in a pre-prepared mobile phase (0.1 mol/L NaNO_3_) to a concentration of 1 mg/mL, then filtered through a 0.22 μm membrane and transferred to a sample bottle for measurement.

#### 2.4.2. Monosaccharide Composition Analysis

The monosaccharide composition of LPS, G-LPS(8), and G-LPS(16) was analyzed using high-performance anion exchange chromatography (HPAEC) on a Thermo ICS 5000+ ion chromatography system (Thermo Fisher Scientific, Waltham, MA, USA) equipped with an electrochemical detector. The specific method referred to the method described by Zhu et al. and was modified slightly [18]. LPS, G-LPS(8), and G-LPS(16) samples (2 mg) and 1 mL of TFA solution (2M) were heated at 121 °C for 2 h. Running the nitrogen and blow drying followed. The sample was then washed with methanol and dried again. This methanol cleaning step was repeated 2–3 times. Finally, the dried sample was dissolved in sterile water and transferred to a chromatographic vial for analysis.

#### 2.4.3. Fourier Transform Infrared (FT-IR) Spectra

The structural properties of LPS, G-LPS(8), and G-LPS(16) were analyzed using a Nicolet iS5 FTIR spectrometer (Thermo Fisher Scientific). The spectra were collected from 4000 to 400 cm^−1^ at a resolution of 2 cm⁻^1^, and 64 scans were performed [19].

### 2.5. Radical Scavenging Activity

The antioxidant activities of LPS, G-LPS(8), and G-LPS(16) were assessed using *in vitro* chemical models, specifically the DPPH and ABTS free radical scavenging assays, with Vitamin C (Vc) serving as a positive control. The specific method referred to the method reported by Ai et al. and slightly modified [20]. Each sample and Vc are configured as a solution of gradient concentration for backup. Then, it was mixed and incubated with a pre-configured DPPH–ethanol solution and ABTS–K_2_S_2_O_8_ mixture, recording its absorbance at 517 nm and 734 nm, respectively, and calculating free radical removal (%) using the following Formula (1):

(1) $$\mathrm{Radical}\;\mathrm{scavenging}\;\mathrm{rate}\;(\%)={(A}_{0}-(A_{s}-A_{x}))/A_{0}\times 100\%$$

where A_0_ refers to the absorbance of the mixture excluding the sample, A_S_ represents the absorbance of the reaction solution, and A_X_ corresponds solely to the absorbance of the sample.

### 2.6. In Vitro Fermentation

#### 2.6.1. Fermentation Medium Preparation

The base medium contained (%) 0.2 NaHCO_3_, 0.01 NaCl, 0.001 MgSO_4_·7H_2_O, 0.004 K_2_HPO_4_, 0.001 CaCl_2_·6H_2_O, 0.004 KH_2_PO_4_, 0.0025 heme chloride, 0.0002 vitamin K1, 0.05 L-cysteine hydrochloride, 0.05 bile salt, 0.2 peptone and 0.2 yeast extract. And 2 mL Tween 80, 4 mL Azurin solution (0.25 g/L) were added. In addition, 0.05 g/L LPS, G-LPS(8) and G-LPS(16) were added to the fermentation medium. All media were sterilized at 121 °C for 15 min.

#### 2.6.2. Fecal Suspension Preparation

Fecal samples for the study were sourced from four healthy volunteers, aged 20–25, with no recent antibiotic use (at least three months). The study received approval from the Nanchang University Ethics Committee, and informed consent was obtained from all participants. Each donor’s stool (5 g) was collected within 15 min and processed anaerobically in a glove box (LAI-3, Shanghai Longyue Instrument Equipment Co., Ltd., Shanghai, China). The feces were diluted with PBS buffer containing L-cysteine hydrochloride, filtered to remove residue, and combined with samples from all four volunteers in equal amounts for use in fermentation inoculation.

#### 2.6.3. *In Vitro* Simulated Fermentation

The experiment was carried out using the methods reported in the literature with slight modifications [13]. A total of 60 sterile anaerobic tubes were divided into four groups: Group A (blank control, CO), Group B (LPS control), Group C (G-LPS(8) sample), and Group D (G-LPS(16) sample). To each anaerobic tube, 1 mL microbial suspension and 9 mL corresponding culture medium were added. These tubes were then fermented in an anaerobic incubator at 37 °C and sampled at five time points of 0, 6, 12, 24, and 48 h, respectively. The fermentation solution was collected and frozen. The experiment adopted a parallel design to ensure that each sample and each time point had three parallels. After fermentation, the fermentation supernatant and bacterial precipitation of each group were harvested by centrifugation for further analysis.

#### 2.6.4. Determination of pH

At the specified sampling time point, 2 mL of fermentation liquid was taken from the anaerobic tube to the 10 mL EP tube to determine the pH value of the fermentation products by a pH meter (FE28, Mettler Toledo, Shanghai, China).

#### 2.6.5. Gut Microbiota Analysis

DNA was extracted from bacterial precipitates that had been fermented for 48 h in each group using the QIAamp Fast DNA Stool Mini Kit. The V3–V4 region of the 16S rRNA gene was amplified by PCR with the primers 338F and 806R. Following PCR amplification, the resulting libraries were sequenced on the Illumina MiSeq platform at Shanghai Majorbio Bio-pharm Technology Co., Ltd. (Shanghai, China). The 16S sequencing reads were then denoised and processed using the DADA2 algorithm in Qiime2 (v2022.2) to generate an amplicon sequence variant (ASV) table. Species annotation was performed using the Silva138/16S_bacteria database. Finally, the data were analyzed using the Majorbio Cloud Platform (www.majorbio.com, accessed on 18 December 2024).

#### 2.6.6. Untargeted Metabolomics Analysis

From each group, 2 mL samples were fermented for 48 h, freeze-dried, and sent to Majorbio for non-targeted metabolomics. The freeze-dried samples were reconstituted in 200 µL of an extract solution (4:1 methanol-to-water ratio, containing four internal standards), then homogenized for 6 min using a frozen tissue grinder (Wonbio-96c, Shanghai Wanbai Biotechnology Co., Ltd., Shanghai, China). Ultrasonic treatment at 40 kHz for 30 min was applied to enhance extraction, followed by a 30 min incubation at −20 °C to promote phase separation. After centrifugation at 13,000× *g* for 10 min at 4 °C, the supernatant was transferred to an injection vial for UPLC-MS/MS analysis (UHPLC-Exploris240, Thermo Fisher Scientific). A combined 20 µL of supernatant from all samples was also pooled into a QC sample vial for quality control. Chromatographic separation was performed using an ACQUITY UPLC HSS T3 column at 40 °C, with a mobile phase of 5% acetonitrile with 0.1% formic acid and 47.5% acetonitrile with 0.1% formic acid mixed with 47.5% isopropyl alcohol. Data analysis, including PCA, Venn analysis, and KEGG pathway analysis, was carried out on the Majorbio Cloud Platform.

### 2.7. Statistical Analysis

Data are expressed as mean ± standard deviation (SD) across three independently tested sample replicates. The data analysis and correlation analysis were performed with SPSS 26.0 software. For group comparisons, univariate analysis of variance was applied followed by the Tukey–Kramer test. *p* < 0.05 was regarded as statistically significant.

## 3. Results and Discussion

### 3.1. Total Sugar, Reducing Sugar, and Molecular Weight of LPS, G-LPS(8), and G-LPS(16)

The total sugar and reducing sugar content of LPS, G-LPS(8), and G-LPS(16) were quantitatively analyzed, and the specific outcomes are presented in Table 1. The analysis revealed no significant difference in total sugar content among G-LPS(8), G-LPS(16), and LPS (*p* > 0.05), indicating that the degradation treatment had little effect on the polysaccharide stability. Regarding the reducing sugar content, in contrast to LPS (1.09 ± 0.05 mg/mL), G-LPS(8) and G-LPS(16) exhibited significant increments, particularly G-LPS(16), whose reducing sugar content reached as high as 3.50 ± 0.12 mg/mL. This implies that the condition of the enhanced enzyme treatment helps to break more glycosidic bonds in the molecular chain of LPS, thereby exposing more reducing ends [21].

Furthermore, the HPGPC elution profiles of LPS, G-LPS(8), and G-LPS(16) are illustrated in Figure S1. Based on the calibration equation obtained through the linear regression of the calibration curve, the mean Mw of these three polysaccharides was determined to be 67.26 kDa, 20.86 kDa, and 7.32 kDa, respectively (Table 1). Prior research has indicated that the metabolic activity of *Bacteroides thetaiotaomicron* (Bt), a common carbohydrate-degrading bacterium in the gut microbiota, diminishes as the Mw of the polysaccharide increases [22]. A comparable study further elucidated that polysaccharides with lower Mw are more advantageous for gut microbial ecology [23]. This suggests that the G-LPS(8) and G-LPS(16) obtained through enzymatic degradation in this study may exhibit enhanced fermentation characteristics and prebiotic potential.

### 3.2. Monosaccharide Composition of LPS, G-LPS(8), and G-LPS(16)

The monosaccharide composition of LPS, G-LPS(8), and G-LPS(16) were summarized in Table 2 and depicted in Figure S2. It was discovered that the composition of these three polysaccharides mainly comprises Ara, Rha, Gal, Glc, and Man. Notably, Glc had the highest proportion, varying between 51.05% and 63.88%, followed by Man, which ranged from 32.48% to 43.85%, while several uronic acid components were essentially undetectable. These findings suggest that LPS and its derivatives (G-LPS(8) and G-LPS(16)) are predominantly composed of neutral sugars, and the monosaccharide composition remains unchanged after enzymatic treatment. This aligns with prior studies [4,5,24]. Importantly, as the amount of enzyme increased, the molar ratio of Glc exhibited a gradual decline, whereas that of Man increased, likely because β-glucanase, a substrate-specific enzyme, acts directly on the α-1, 6-glucosidic bonds in LPS, leading to the depolymerization of glucan chains into smaller Mw polymers [25]. This phenomenon also elucidates why G-LPS(8) and G-LPS(16) exhibit lower Mw and total sugar content compared to LPS (Table 1), as the small molecular fragments generated by glucanase degradation are eliminated during dialysis. On the other hand, Wang et al. highlighted that the monosaccharide composition and relative proportions of polysaccharides significantly influence their biological activities [26]. In this study, the progressively increasing ratios of Man, Rha, Gal, and Ara, while the proportion of Glc decreases, may potentially affect the antioxidant and prebiotic activities of LPS.

### 3.3. FT-IR Spectra Analysis

**Figure 1:**
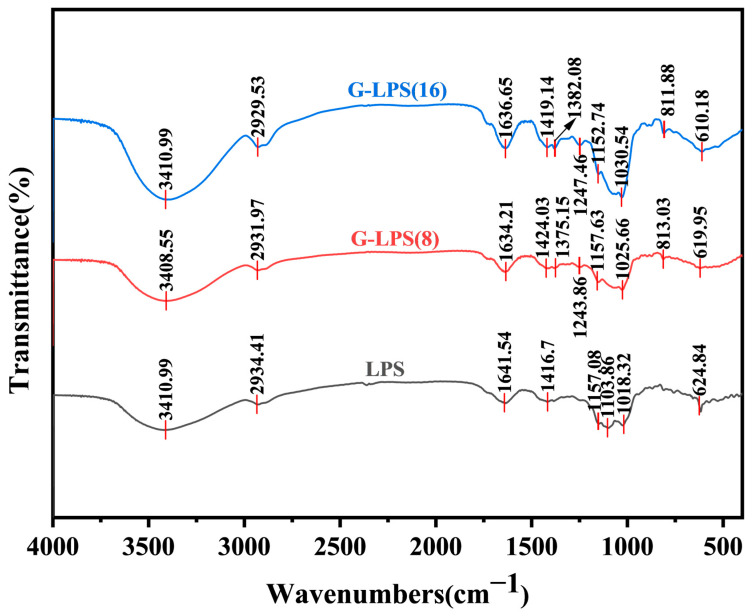
FT-IR spectra of LPS, G-LPS(8), and G-LPS(16) in the range of 4000–400 cm^−1^. LPS represents the original *Lilium* polysaccharide, and G-LPS(8) and G-LPS(16) are degradation products of LPS treated with 0.8 × 10^3^ U/g and 1.6 × 10^3^ U/g β-glucanase, respectively.

The typical FT-IR spectra of LPS, G-LPS(8), and G-LPS(16) were shown in Figure 1. Both LPS and its enzymatically degraded derivatives, G-LPS(8) and G-LPS(16), display comparable characteristic absorption peaks within the wavenumber range of 4000 to 400 cm^−1^, suggesting that the degradation process has not altered the primary functional group structures of the polysaccharides. It is worth noting that the prominent and broad absorption peak around 3410 cm⁻^1^ is indicative of the O-H bond stretching vibrations in the sugar residues [27]. The absorption peaks observed around 2930, 1635, and 1420 cm^−1^ correspond to the stretching vibrations of the C-H bond, C=O bond, and C-O bond in the sugar ring, respectively [28]. Additionally, a range of weaker absorption peaks observed between 1000 and 1200 cm⁻^1^ indicates the presence of pyranoid rings [20]. Importantly, when compared to LPS, G-LPS(8) and G-LPS(16) exhibit additional absorption peaks at 1375.15 cm^−1^ and 1382.08 cm^−1^ for symmetric C-H bending vibrations of methyl groups, as well as at 1243.86 cm^−1^ and 1247.46 cm^−1^ for C-O vibrations of acetyl groups [29]. These changes could be attributed to the cleavage of glycosidic bonds induced by enzymatic treatment, leading to the exposure of these functional groups. Likewise, the characteristic absorption peaks at 813.03 cm^−1^ and 811.88 cm^−1^ in the two degradation products serve as distinctive markers of glucan [19]. Overall, despite the fact that the fundamental infrared spectral characteristics of LPS largely remained intact post-enzymatic hydrolysis, the emergence of certain new absorption peaks revealed that enzymatic hydrolysis could lead to the exposure of more internal groups.

### 3.4. Antioxidant Activity of LPS, G-LPS(8) and G-LPS(16)

**Figure 2:**
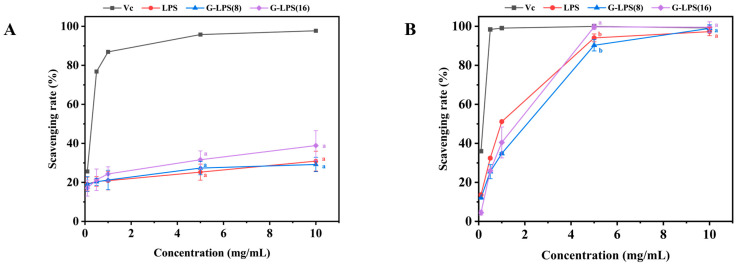
Changes in antioxidant capacity. Including the scavenging activities of LPS, G-LPS(8), G-LPS(16), and V_C_ on (**A**) DPPH (**B**) and ABTS free radicals at different concentrations. LPS represents the original *Lilium* polysaccharide, and G-LPS(8) and G-LPS(16) are degradation products of LPS treated with 0.8 × 10^3^ U/g and 1.6 × 10^3^ U/g β-glucanase, respectively. In the same column, distinct lowercase letters represent statistically significant differences between treatments (*p* < 0.05).

The antioxidant activities of LPS, G-LPS(8), and G-LPS(16) are summarized in Figure 2. All three polysaccharides demonstrated significant, dose-dependent effects on DPPH free radical scavenging within concentrations ranging from 0.1 to 10 mg/mL (Figure 2A). Notably, G-LPS(8) exhibited superior DPPH scavenging ability compared to LPS at concentrations between 0.5 and 5 mg/mL. G-LPS(16) showed even greater enhancement, outperforming LPS over a wider concentration range (0.5 to 10 mg/mL). Similarly, all three polysaccharides also displayed notable ABTS free radical scavenging activity, following a dose-dependent trend (Figure 2B). At 5 mg/mL, LPS achieved a 94.06% ABTS scavenging rate, while G-LPS(16) reached 99.72%, comparable to vitamin C. At 10 mg/mL, G-LPS(8) also demonstrated a high scavenging rate of 98.95%.

The experimental data presented above indicate that enzymatic hydrolysis could enhance the scavenging activity of LPS against DPPH and ABTS free radicals to a certain extent. This enhancement was attributed to the effective reduction in the Mw of LPS during the enzymatic hydrolysis process. Polysaccharides with lower Mw exhibited greater reactivity with free radicals due to their higher reducing hydroxyl content and larger specific surface area, thus, demonstrating stronger antioxidant properties [30,31]. These findings align with previous studies by Tian and Wu et al., which showed that enzymatic hydrolysis can improve the antioxidant capacity of *Gracilariopsis lemaneiformis* and *Auricularia auricula* polysaccharides by significantly reducing their Mw [32,33]. Additionally, growing evidence suggests that polysaccharides, as natural antioxidants, can mitigate oxidative damage by scavenging free radicals, thereby modulating gut microbiota and enhancing gut health [34]. Consequently, boosting the antioxidant activity of polysaccharides is anticipated to not only improve their capacity to regulate intestinal homeostasis but also offer substantial support for the development of novel intestinal health supplements. In summary, G-LPS(16), which exhibited superior antioxidant activity in this study, holds considerable potential for alleviating intestinal oxidative stress and are promising candidates for use as effective antioxidant supplements, with broad applications in promoting intestinal health.

### 3.5. Effects of In Vitro Fermentation on pH of LPS, G-LPS(8), and G-LPS(16)

**Figure 3:**
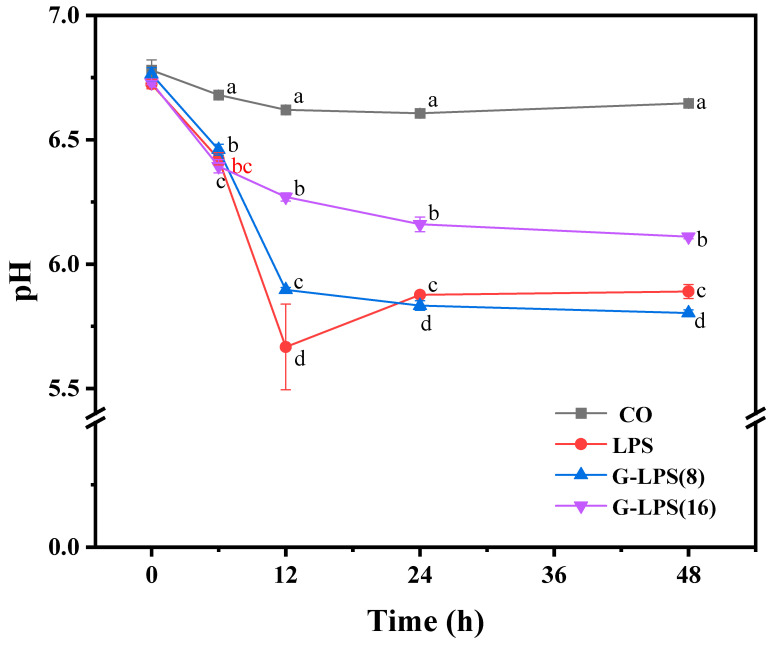
Changes in pH value during fermentation. In the same column, distinct lowercase letters represent statistically significant differences between treatments (*p* < 0.05). LPS represents the original *Lilium* polysaccharide, and G-LPS(8) and G-LPS(16) are degradation products of LPS treated with 0.8 × 10^3^ U/g and 1.6 × 10^3^ U/g β-glucanase, respectively.

Throughout the entire process of simulated intestinal fermentation *in vitro*, the acidic byproducts generated by intestinal microbial metabolism had a substantial impact on the pH value in the colon. This could serve as a crucial index for evaluating the extent of polysaccharide utilization by the microbial community. As depicted in Figure 3, at the first 12 h, the pH in the ferments of the CO, LPS, G-LPS(8), and G-LPS(16) groups declined rapidly. This might be attributed to the rapid growth of microorganisms in the logarithmic phase and the production of copious amounts of organic acids [35]. Subsequently, the pH of LPS started to rise after 12 h of fermentation. After 24 h, the pH of CO also exhibited an upward trend, which might be associated with the further utilization of metabolites, such as short-chain fatty acids (SCFAs), by intestinal microorganisms [36]. Interestingly, the pH values of G-LPS(8) and G-LPS(16) groups consistently decreased during the whole fermentation process and reached 5.80 and 6.11 at 48 h, respectively. Previous studies have shown that a slightly acidic environment with a low pH (5.5~6.5) is conducive to the increase in butyric acid production, which in turn has a positive impact on the health of the intestinal environment [37]. In summary, G-LPS(8) and G-LPS(16) obtained by enzymatic hydrolysis in this study may further maintain the healthy microecology of the gut by stimulating the production of butyric acid and have a positive impact on the proliferation of specific microbial communities and the production of metabolites.

### 3.6. Effects of In Vitro Fermentation on Gut Microbial Diversity of LPS, G-LPS(8), and G-LPS(16)

**Figure 4:**
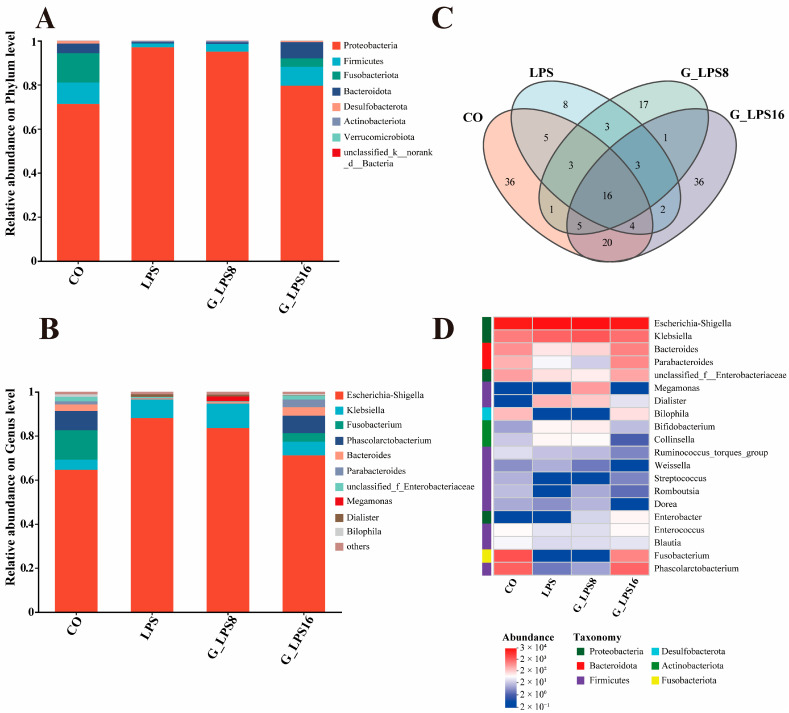
Structure of gut microbial community after 48 h *in vitro* fermentation. (**A**) Histograms of relative species abundance at the phylum level, (**B**) histograms of relative species abundance at the genus level, (**C**) Venn plots of gut microbiota, and (**D**) heatmap analysis of gut microbial community structure at the genus level for different samples. CO was fermented in blank control medium for 48 h; LPS, G_LPS8, and G_LPS16 were fermented in medium supplemented with LPS, G-LPS(8), and G-LPS(16) for 48 h, respectively.

The human gut microbiota is essential for maintaining overall health, particularly in enhancing energy metabolism and stabilizing the immune system [38]. In this study, the intestinal flora structure of LPS and its enzymolytic derivatives (G-LPS(8) and G-LPS(16)) after 48 h of *in vitro* fermentation was shown in Figure 4. In Table 3 and Figure 4C, the ASV analysis and α diversity indices (Chao 1, Simpson, and Shannon) indicate that LPS, G-LPS(8), and G-LPS(16) can influence the structural composition of fecal microbiota to varying extents.

Furthermore, Figure 4A,B depict the alterations in the distribution of intestinal microorganisms at the phylum and genus levels after 48 h of fermentation, respectively. *Bacteroidetes*, a prominent group of carbohydrate-degrading bacteria in the colon, are capable of breaking down indigestible polysaccharides by producing polysaccharide lyases and glycosidases. This process generates beneficial metabolites such as SCFAs, which help prevent gastrointestinal diseases and inflammation [39]. In this study, a higher proportion of *Bacteroidetes* (7.32%) was observed in the fermentation broth supplemented with G-LPS(16) compared to the CO group (4.34%). *Bacteroides*, a member of *Bacteroidetes*, has been recognized as a key microorganism in degrading dietary carbohydrates in the human colon [40]. Research has demonstrated that specific species, such as *Bacteroides fragilis* and *Bacteroides ovale*, can protect the gut from inflammatory diseases [41]. Our findings revealed that among the three polysaccharide groups, low-molecular-weight G-LPS(16) was more effective in promoting the proliferation of *Bacteroides*. Additionally, the relative abundance of *Parabacteroides* was greater in the G-LPS(16) group (3.41%) compared to the CO group (1.36%).

For *Firmicutes*, specific members within this phylum in the gut are capable of producing SCFAs, predominantly butyrate, through the fermentation of non-digestible carbohydrates [42]. In the current investigation, the relative abundance of *Firmicutes* was observed to incrementally rise with the reduction in the Mw of polysaccharides. Furthermore, in the LPS- and G-LPS(8)-supplemented groups, the lower pH noted at the conclusion of fermentation (Figure 3) likely exerts a substantial impact on the gut microbiota, as the acidic environment promotes the proliferation of *Firmicutes* while suppressing the growth of *Bacteroidetes* [43]. Conversely, prior research has documented an elevated *Firmicutes*-to-*Bacteroidetes* ratio in the microbiomes of individuals suffering from diabetes and obesity [44,45]. Within this experimental framework, the *Firmicutes*-to-*Bacteroidetes* ratio in the G-LPS(16) group diminished as the relative abundance of *Bacteroidetes* escalated. This observation suggests that G-LPS(16) might exert a beneficial influence on the management of diabetes. *Phascolarctobacterium*, a representative genus of *Firmicutes* and a key probiotic in the human gut, regulates host glycogen metabolism by metabolizing succinic acid, thereby mitigating the risk of *Clostridium* difficile infection [46]. Our findings indicate a marked enhancement in the relative abundance of *Phascolarctobacterium* in the G-LPS(16) group when compared to the other two polysaccharide groups. Moreover, *Megamonas*, a *Firmicutes* species commonly found in human feces, possesses a gene cluster that encodes for secretory endoglucanase, which plays a crucial role in the fermentation and utilization of polysaccharides as well as the coordinated transport of sugars [47], and it contributes to the synthesis of propionate. This organism has been shown to positively affect the alleviation of metabolic dysfunction-associated fatty liver disease (MAFLD) [38,48]. In this study, the relative abundance of *Megamonas* in the G-LPS(8) group reached 2.20%, marking a significant increase relative to other groups, indicating that G-LPS(8) facilitates the growth of this beneficial bacterium.

*Proteobacteria*, which include genera such as *Escherichia-Shigella*, *Salmonella*, *Klebsiella*, etc., are facultative anaerobes that can disturb the equilibrium of the gut microbiota [49]. In the present study, the abundance of *Proteobacteria* was higher in the groups treated with LPS, G-LPS(8), and G-LPS(16) when compared to the CO group. Interestingly, a progressive reduction in the relative abundance of *Proteobacteria* was observed as the Mw of the polysaccharides decreased. This suggests that reducing the Mw of LPS can effectively enhance their inhibitory effect on harmful bacteria such as *Proteobacteria*. The primary genera of *Proteobacteria* identified in this study were *Escherichia-Shigella* and *Klebsiella*. It was observed that the relative abundance of these genera increased in the LPS, G-LPS(8), and G-LPS(16) groups, possibly because these bacteria can utilize oligosaccharides generated from the degradation of LPSs as a nutrient source to sustain their growth in the fermentation culture environment [50]. However, as the Mw of the polysaccharides decreased, the growth of *Escherichia-Shigella* was notably suppressed, likely due to the accumulation of fermentation byproducts such as SCFAs, which are produced by beneficial gut microorganisms, like *Bacteroidetes*. Moreover, previous studies have highlighted a link between higher levels of *Fusobacteria* and the onset of gastric cancer [51]. In the present study, after 48 h of fermentation, the relative abundance of *Fusobacterium* in the LPS, G-LPS(8), and G-LPS(16) groups was significantly reduced compared to the blank group. Additionally, at the genus level, all three polysaccharide groups showed a notable decrease in *Fusobacterium* abundance when compared to the CO group.

The heatmap displays the relative abundance of the top 20 bacterial genera across different groups (Figure 4D). After 48 h of fermentation, an increase in the abundance of beneficial bacteria, such as *Bacteroides*, was observed, while harmful bacteria like *Fusobacterium* showed varying degrees of reduction in the groups treated with LPS and its hydrolyzed derivatives. Slight variations in the bacterial patterns were observed among the polysaccharides degraded by different enzyme addition levels. For instance, the G-LPS(8) group exhibited a higher abundance of *Megamonas*, whereas the G-LPS(16) group predominantly supported the growth of *Bacteroides*, *Parabacteroides*, and *Phascolarctobacterium*. This mechanism of action differs somewhat from the industry-recognized effects of prebiotic inulin, which, according to Teferra et al., primarily exerts its probiotic activity by stimulating the proliferation of beneficial bacteria such as *Lactobacillus* [52]. Overall, the gut microbiome analysis suggests that LPS supplementation influences microbial composition in a structure-dependent manner, potentially playing a role in maintaining intestinal homeostasis.

### 3.7. Effects of In Vitro Fermentation on Gut Bacteria Fermentation Metabolites of LPS, G-LPS(8), and G-LPS(16)

#### 3.7.1. Screening and Identification of Differential Metabolites

**Figure 5:**
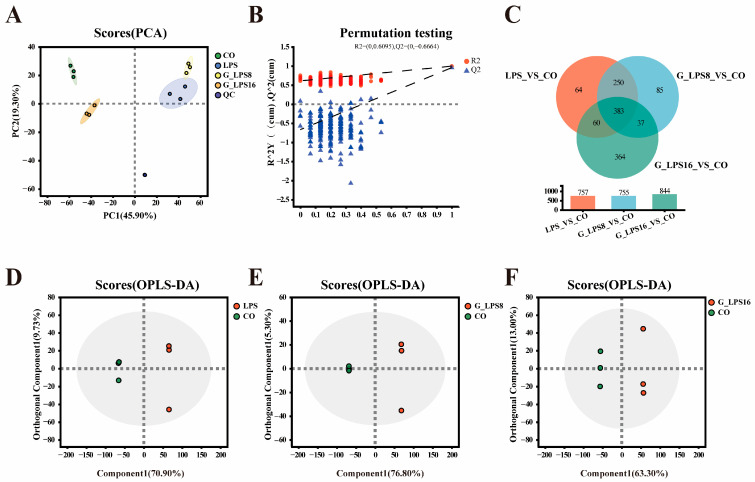
Metabolite changes induced by LPS, G-LPS(8), and G-LPS(16) after 48 h *in vitro* fermentation. (**A**) PCA analysis of metabolomics data. Different groups are represented by different colors. (**B**) PLS-DA replacement test. Q2 value < 0.05 indicates that the model is robust and reliable without overfitting. (**C**) Venn diagram of differentiated metabolites between groups. OPLS-DA scores of (**D**) LPS, (**E**) G-LPS(8), (**F**) G-LPS(16), and CO groups. CO was fermented in blank control medium for 48 h; LPS, G_LPS8, and G_LPS16 were fermented in medium supplemented with LPS, G-LPS(8), and G-LPS(16) for 48 h, respectively.

To further elucidate the potential effects of LPS and its enzymatically degraded derivatives on intestinal microbial metabolites, an off-target metabolomics approach was employed to comprehensively analyze the metabolites in the fecal bacterial community fermentation supernatant. The PCA score plot (Figure 5A) demonstrates a significant separation between the CO group and the LPS, G-LPS(8), and G-LPS(16) groups under a mixed ion detection mode (a combination of positive and negative ion modes). Additionally, the permutation test results of PLS-DA (Figure 5B) confirmed the robust adaptability and predictive performance of the model. OPLS-DA also revealed significant metabolic differences between the LPS-treated group and the CO group across different Mws (Figure 5D–F). Furthermore, metabolites with a VIP value > 1, *p* < 0.05, and fold change (FC) threshold of 1, as determined by the OPLS-DA model, were defined as significantly different metabolites. Compared to the control group, 757, 755, and 844 significantly different metabolites were identified in the LPS, G-LPS(8), and G-LPS(16) treatment groups, respectively (Figure 5C).

**Figure 6:**
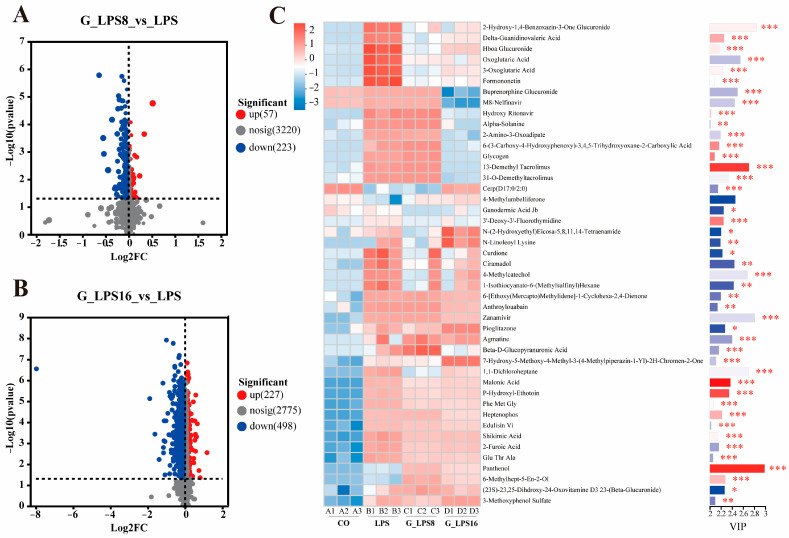
Volcanic maps of metabolite differences between (**A**) G-LPS(8), (**B**) G-LPS(16), and LPS groups after 48 h *in vitro* fermentation. (**C**) Heatmap of differential metabolites (VIP ≥ 2, *p* < 0.05, with FC ≥ 1). On the right, a bar chart displays the VIP values of metabolites, where the length of each bar reflects the metabolite’s contribution to the observed differences between the two groups, with a minimum value of 1. Larger values correspond to greater differences. The color of each bar represents the significance of the metabolite differences, with darker colors indicating smaller *p*-values and, consequently, more significant differences. * *p* < 0.05, ** *p* < 0.01, *** *p* < 0.001. CO was fermented in blank control medium for 48 h; LPS, G_LPS8, and G_LPS16 were fermented in medium supplemented with LPS, G-LPS(8), and G-LPS(16) for 48 h, respectively.

Volcano plots indicated that 57 metabolites were significantly upregulated, while 223 metabolites were downregulated in the G-LPS(8) group relative to the LPS group. In contrast, the G-LPS(16) group, with a lower Mw, exhibited a broader range of metabolites (Figure 6A,B). Further analysis was conducted on metabolites with a VIP ≥ 2. As shown in Figure 6C and Table S1, the changes in metabolites were primarily distributed among organic acids and derivatives (24.44%), organic oxygen compounds (15.56%), organoheterocyclic compounds (8.89%), phenylpropanoids and polyketides (13.33%), lipids and lipid-like molecules (13.33%), and benzenoids (6.67%). Compared to the CO group, LPS supplementation predominantly influenced organic acids and their derivatives, organic oxygen compounds, and certain phenylpropanoids and polyketides, such as oxoglutaric acid, glycogen, and alpha-solanine. G-LPS(8) affected metabolites similarly to LPS, albeit with slight variations in the extent of the effects. In the G-LPS(16) group, some organic acids and derivatives (e.g., 3-methoxyphenol sulfate, N-(2-hydroxyethyl)eicosa-5,8,11,14-tetraenamide), phenylpropanoids and polyketides, and benzenoids (e.g., pioglitazone) increased, while other organic acids and derivatives (e.g., M8-nelfinavir) and organoheterocyclic compounds (e.g., buprenorphine glucuronide) decreased. To sum up, LPS and its hydrolyzed derivatives can alter microbial metabolism to varying degrees. It is also worth noting that as enzymatic hydrolysis intensified, we detected more differential metabolites, which may be caused by the lower Mw displayed and the exposure of more internal groups.

#### 3.7.2. Pathway Enrichment Analysis

**Figure 7:**
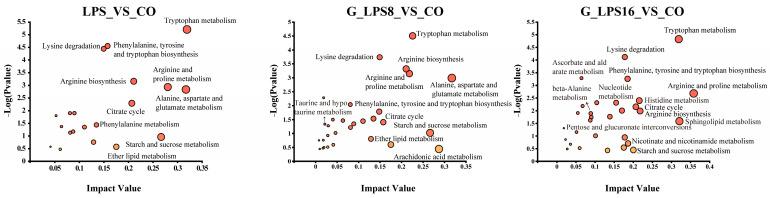
Remarkably changed KEGG pathways among CO, LPS, G-LPS(8), and G-LPS(16) groups after 48 h *in vitro* fermentation. CO was fermented in blank control medium for 48 h; LPS, G_LPS8, and G_LPS16 were fermented in medium supplemented with LPS, G-LPS(8), and G-LPS(16) for 48 h, respectively.

The KEGG database was utilized to perform an in-depth predictive analysis of metabolic pathways, with the findings presented in Figure 7. When compared to the CO group, the three polysaccharide treatment groups (LPS, G-LPS(8), and G-LPS(16)) primarily influenced amino acid metabolism. This encompasses pathways such as tryptophan metabolism, alanine, aspartate, and glutamate metabolism, arginine and proline metabolism, phenylalanine, tyrosine, and tryptophan biosynthesis, lysine degradation, phenylalanine metabolism, and D-amino acid metabolism, among others. In the LPS, G-LPS(8), and G-LPS(16) groups, 5-hydroxyindoleacetic acid levels in tryptophan metabolism and oxoglutaric acid levels in alanine, aspartate, and glutamate metabolism were significantly elevated, with the magnitude of increase inversely related to the Mw. Similarly, putrescine levels in arginine and proline metabolism in the LPS and G-LPS(16) groups, and phenylacetic acid levels in phenylalanine metabolism in the G-LPS(8) and G-LPS(16) groups, exhibited a similar increasing trend.

Moreover, the LPS, G-LPS(8), and G-LPS(16) treatments also impacted carbohydrate metabolism. For instance, citric acid levels in the citrate cycle (TCA cycle) and galactinol levels in galactose metabolism were variably increased across the LPS, G-LPS(8), and G-LPS(16) groups. Additionally, butyric acid metabolism was markedly enhanced in the LPS and G-LPS(8) groups, with higher concentrations observed in the G-LPS(8) group relative to the LPS group. This is consistent with the above results that the relative abundance of firmicutes in G_LPS(8) group is higher than LPS. Glycochenodeoxycholic acid 3-glucuronide, involved in the interconversion of pentose and glucuronic acid, was significantly elevated in the G-LPS(16) group. These observations suggest that, when LPS and its derivatives serve as the sole carbon source, they can activate carbohydrate metabolism pathways, such as TCA cycle, galactose metabolism, butyric acid metabolism, and pentose and glucuronic acid interconversion, thereby facilitating fermentation to varying extents.

Furthermore, with decreasing Mw, the number of significantly altered metabolic pathways increased in the G-LPS(16) group. Notable examples include sphingolipid metabolism (sphingosine), histidine metabolism, nucleotide metabolism (adenosine, guanine, thymine, hypoxanthine), beta-alanine metabolism (spermidine), and ascorbic acid and aldose metabolism. Levels of sphingosine and spermidine were significantly elevated in the G-LPS(16) group, with concentrations markedly higher than those in the LPS and G-LPS(8) groups. Sphingosine, a type of long-chain sphingolipid, has shown antimicrobial properties against a range of pathogens, including *Pseudomonas aeruginosa*, *Staphylococcus aureus*, and *Escherichia coli*, etc. [53]. Spermidine, an aliphatic amine containing several amino groups, is essential for cell growth and proliferation, and it also impacts a wide range of intricate physiological functions, such as enhancing antioxidant activity, modulating ion channels, regulating protein translation, apoptosis, autophagy, and immune responses [54].

In summary, this study demonstrates that LPS and its derivatives exert a substantial influence on the metabolic activities of intestinal microorganisms, primarily through the modulation of amino acid metabolism and carbohydrate metabolism pathways. The structural changes induced by enzymatic hydrolysis, particularly the significant reduction in Mw, may be a critical factor influencing metabolic regulation.

## 4. Conclusions

In this study, two types of *Lilium* polysaccharide degradation products with distinct molecular weights were successfully prepared through β-glucanase treatment, designated as G-LPS(8) and G-LPS(16). The physical and chemical properties, *in vitro* antioxidant capacity, and glycolytic properties of these degradation products were subsequently evaluated and analyzed systematically. Post-enzymolysis, the reducing sugar content of both G-LPS(8) and G-LPS(16) was markedly elevated, and their molecular weights were significantly decreased compared to those prior to treatment, indicating that the enzymolysis process effectively facilitated the structural deconstruction of the polysaccharides. Remarkably, despite enzymatic hydrolysis, the primary infrared spectral structural characteristics and monosaccharide compositions of these two degradation products remained largely unchanged, suggesting that enzymatic hydrolysis can alter the physical properties of polysaccharides while maintaining their fundamental chemical structure. Furthermore, this study observed that the antioxidant activity of LPS after enzymolysis increased in a specific concentration range, indicating that the degradation products possess potential advantages in terms of antioxidant functionality. Additionally, *in vitro* fermentation experiments coupled with multi-omics analysis demonstrated that both LPS and its degradation products exhibited prebiotic effects by selectively modulating intestinal microbial communities. Specifically, G-LPS(16) displayed a higher prebiotic potential, capable of significantly increasing the abundance of beneficial bacteria and inhibiting the proliferation of harmful bacteria. Metabolomic analysis revealed that LPSs with varying molecular weights exerted comparable promoting effects on multiple amino acid and carbohydrate metabolic pathways. Importantly, with the reduction in molecular weight, G-LPS(16) also remarkably stimulated sphingolipid metabolism, nucleotide metabolism, as well as ascorbic acid and uronic acid metabolism, leading to the significant increase in specific metabolites such as sphingosine. These findings offer novel insights into the interplay between colon fermentation properties and the structure of *Lilium* polysaccharides, providing robust evidence for the further development of *Lilium* polysaccharides as potential prebiotics. Although the *in vitro* fermentation results we obtained were positive, the model had some limitations, such as nutritional limitations, which were only suitable for short-term studies, and they could not fully simulate the interaction between polysaccharides and gut microbes *in vivo*. Therefore, further studies are needed to verify the effect *in vivo*.

## Figures and Tables

**Table 1:** Total sugar, reducing sugar content, and relative Mw of LPS, G-LPS(8), and G-LPS(16). LPS represents the original *Lilium* polysaccharide, and G-LPS(8) and G-LPS(16) are degradation products of LPS treated with 0.8 × 10^3^ U/g and 1.6 × 10^3^ U/g β-glucanase, respectively.

	Total Sugar (mg/mL)	Reducing Sugar (mg/mL)	Mw (kDa)
LPS	9.03 ± 0.14 ^a^	1.09 ± 0.05 ^c^	67.26
G-LPS(8)	8.85 ± 0.19 ^a^	1.55 ± 0.14 ^b^	20.86
G-LPS(16)	8.67 ± 0.20 ^a^	3.50 ± 0.12 ^a^	7.32

Notes: In the same column, distinct lowercase letters represent statistically significant differences between treatments (*p* < 0.05).

**Table 2:** Monosaccharide composition of LPS, G-LPS(8), and G-LPS(16). LPS represents the original *Lilium* polysaccharide, and G-LPS(8) and G-LPS(16) are degradation products of LPS treated with 0.8 × 10^3^ U/g and 1.6 × 10^3^ U/g β-glucanase, respectively. The content of each monosaccharide is calculated by molar mass ratio.

	LPS	G-LPS(8)	G-LPS(16)
Rha (%)	1.24	1.40	1.72
Ara (%)	0.43	0.51	0.63
Gal (%)	1.97	2.00	2.76
Glc (%)	63.88	59.56	51.05
Man (%)	32.48	36.52	43.85

**Table 3:** Effects of addition of LPS, G-LPS(8), and G-LPS(16) on α diversity index of gut microbial after 48 h *in vitro* fermentation. CO was fermented in blank control medium for 48 h; LPS, G_LPS8, and G_LPS16 were fermented in medium supplemented with LPS, G-LPS(8), and G-LPS(16) for 48 h, respectively.

Sample	Community Richness	Community Diversity	
	Chao	Simpson	Shannon
CO	90	0.231803	2.129118
LPS	44	0.416559	1.259366
G_LPS8	49	0.376289	1.440898
G_LPS16	87.75	0.272507	1.953527

## Data Availability

The original contributions presented in the study are included in the article/Supplementary Material, further inquiries can be directed to the corresponding authors.

## Supplementary Materials

The following supporting information can be downloaded at: https://www.mdpi.com/article/10.3390/foods14020246/s1, Figure S1. HPGPC elution chromatogram of LPS, G-LPS(8), and G-LPS(16). LPS represents the original *Lilium* polysaccharide, and G-LPS(8) and G-LPS(16) are degradation products of LPS treated with 0.8 × 10^3^ U/g and 1.6 × 10^3^ U/g β-glucanase, respectively. Figure S2. HPAEC chromatograms of standard monosaccharides and LPS, G-LPS(8), and G-LPS(16). LPS represents the original *Lilium* polysaccharide, and G-LPS(8) and G-LPS(16) are degradation products of LPS treated with 0.8 × 10^3^ U/g and 1.6 × 10^3^ U/g β-glucanase, respectively. The peaks of 1–8 were standards: (1) Fuc, (2) Rha, (3) Ara, (4) Gal, (5) Glc, (6) Xyl, (7) Man, (8) Fru, (9) Rib, (10) Gal-UA, (11) Gul-UA, (12) Glc-UA, (13) Man-UA. Table S1: Differential metabolites in VIP cluster heatmaps (VIP ≥ 2, *p* < 0.05, with FC ≥ 1).

## References

[1] Li S., Bao F., Cui Y. (2021). Immunoregulatory Activities of the Selenylated Polysaccharides of *Lilium davidii* var. *unicolor Salisb* In Vitro and In Vivo. Int. Immunopharmacol..

[2] Hou R., Chen J., Yue C., Li X., Liu J., Gao Z., Liu C., Lu Y., Wang D., Li H. (2016). Modification of Lily Polysaccharide by Selenylation and the Immune-Enhancing Activity. Carbohydr. Polym..

[3] Zhang T., Gao J., Jin Z.-Y., Xu X.-M., Chen H.-Q. (2014). Protective Effects of Polysaccharides from *Lilium lancifolium* on Streptozotocin-Induced Diabetic Mice. Int. J. Biol. Macromol..

[4] Hui H., Li X., Jin H., Yang X., Xin A., Zhao R., Qin B. (2019). Structural Characterization, Antioxidant and Antibacterial Activities of Two Heteropolysaccharides Purified from the Bulbs of *Lilium davidii* var. *unicolor Cotton*. Int. J. Biol. Macromol..

[5] Chen Z.-G., Zhang D.-N., Zhu Q., Yang Q.-H., Han Y.-B. (2014). Purification, Preliminary Characterization and in Vitro Immunomodulatory Activity of Tiger Lily Polysaccharide. Carbohydr. Polym..

[6] Bai G., Ye M., Yu L., Yang M., Wang Y., Chen S. (2024). Purification, Characterization, Simulated Gastrointestinal Digestion and Gut Microbiota Fermentation of a Bifidobacterium-Directed Mannoglucan from *Lilium brownii* var. Viridulum. Food Chem. X.

[7] Jiao X., Li F., Zhao J., Wei Y., Zhang L., Yu W., Li Q. (2023). The Preparation and Potential Bioactivities of Modified Pectins: A Review. Foods.

[8] Hu B., Zhang S., Wang Z., Han Q., Zhang D., Zheng Y., Zheng K., Jing Y. (2024). Degradation Method, Structural Characteristics, Biological Activity and Structure-Activity Relationship of Degraded Polysaccharides. Food Rev. Int..

[9] Ma G., Xu Q., Du H., Muinde Kimatu B., Su A., Yang W., Hu Q., Xiao H. (2022). Characterization of Polysaccharide from *Pleurotus eryngii* during Simulated Gastrointestinal Digestion and Fermentation. Food Chem..

[10] Ding Q., Nie S., Hu J., Zong X., Li Q., Xie M. (2017). In Vitro and in Vivo Gastrointestinal Digestion and Fermentation of the Polysaccharide from *Ganoderma atrum*. Food Hydrocoll..

[11] Wang Y., Liu J., Chen L., Jin S., An C., Chen L., Yang B., Schols H.A., De Vos P., Bai W. (2023). Effects of Thermal Treatments on the Extraction and in Vitro Fermentation Patterns of Pectins from Pomelo (*Citrus grandis*). Food Hydrocoll..

[12] Zhao S., Lau R., Chen M.-H. (2024). Influence of Chain Length on the Colonic Fermentation of Xylooligosaccharides. Carbohydr. Polym..

[13] Sun X., Yang Y., Song C., Ai C., Yang J., Song S. (2024). Degradation of Low-Molecular-Weight Fucoidans by Human Intestinal Microbiota and Their Regulation Effect on Intestinal Microbiota and Metabolites during in Vitro Fermentation. Food Biosci..

[14] Zhao S., Yun J., Kang Y., Yang P., Cheng Y., Qiao J., Niu J., Zhang L. (2024). Emulsifying and Ethanol-Induced Gelling Properties of Capsicum Pectin: The Impact of Capsicum Variety and Deproteinization Using the Sevag Method. Int. J. Biol. Macromol..

[15] Chen W., Gao L., Song L., Sommerfeld M., Hu Q. (2023). An Improved Phenol-Sulfuric Acid Method for the Quantitative Measurement of Total Carbohydrates in Algal Biomass. Algal Res..

[16] Prasertsung I., Chutinate P., Watthanaphanit A., Saito N., Damrongsakkul S. (2017). Conversion of Cellulose into Reducing Sugar by Solution Plasma Process (SPP). Carbohydr. Polym..

[17] Hou Y., Zhao J., Yin J., Nie S. (2023). Structural Properties of *Bletilla striata* Polysaccharide and the Synergistic Gelation of Polysaccharide and Xanthan Gum. Food Hydrocoll..

[18] Zhu M., Huang R., Wen P., Song Y., He B., Tan J., Hao H., Wang H. (2021). Structural Characterization and Immunological Activity of Pectin Polysaccharide from Kiwano (*Cucumis metuliferus*) Peels. Carbohydr. Polym..

[19] Hui H., Jin H., Li X., Yang X., Cui H., Xin A., Zhao R., Qin B. (2019). Purification, Characterization and Antioxidant Activities of a Polysaccharide from the Roots of *Lilium davidii* var. *unicolor Cotton*. Int. J. Biol. Macromol..

[20] Ai J., Yang Z., Liu J., Schols H.A., Battino M., Bao B., Tian L., Bai W. (2022). Structural Characterization and In Vitro Fermentation Characteristics of Enzymatically Extracted Black Mulberry Polysaccharides. J. Agric. Food Chem..

[21] Fan L., Zhu X., Zhang D., Li D., Zhang C. (2024). In Vitro Digestion Properties of Laiyang Pear Residue Polysaccharides and It Counteracts DSS-Induced Gut Injury in Mice via Modulating Gut Inflammation, Gut Microbiota and Intestinal Barrier. Int. J. Biol. Macromol..

[22] Wong J.P.H., Chillier N., Fischer-Stettler M., Zeeman S.C., Battin T.J., Persat A. (2024). *Bacteroides thetaiotaomicron* Metabolic Activity Decreases with Polysaccharide Molecular Weight. mBio.

[23] Wu D.-T., He Y., Yuan Q., Wang S., Gan R.-Y., Hu Y.-C., Zou L. (2022). Effects of Molecular Weight and Degree of Branching on Microbial Fermentation Characteristics of Okra Pectic-Polysaccharide and Its Selective Impact on Gut Microbial Composition. Food Hydrocoll..

[24] Li W., Wang Y., Wei H., Zhang Y., Guo Z., Qiu Y., Wen L., Xie Z. (2020). Structural Characterization of Lanzhou Lily (*Lilium davidii* var. *unicolor*) Polysaccharides and Determination of Their Associated Antioxidant Activity. J. Sci. Food Agric..

[25] Wang X., Zhang Y., Li M., Qin Q., Xie T. (2022). Purification and Characterization of Dextranase from *Penicillium cyclopium* CICC-4022 and Its Degradation of Dextran. Int. J. Biol. Macromol..

[26] Wang Z., Zheng Y., Lai Z., Hu X., Wang L., Wang X., Li Z., Gao M., Yang Y., Wang Q. (2024). Effect of Monosaccharide Composition and Proportion on the Bioactivity of Polysaccharides: A Review. Int. J. Biol. Macromol..

[27] Song S., Wu S., Ai C., Xu X., Zhu Z., Cao C., Yang J., Wen C. (2018). Compositional Analysis of Sulfated Polysaccharides from Sea Cucumber (*Stichopus japonicus*) Released by Autolysis Reaction. Int. J. Biol. Macromol..

[28] Gao J. (2015). Structural Characterisation, Physicochemical Properties and Antioxidant Activity of Polysaccharide from *Lilium lancifolium* thunb. Food Chem..

[29] Shi X.-D., Nie S.-P., Yin J.-Y., Que Z.-Q., Zhang L.-J., Huang X.-J. (2017). Polysaccharide from Leaf Skin of *Aloe barbadensis* miller: Part I. Extraction, Fractionation, Physicochemical Properties and Structural Characterization. Food Hydrocoll..

[30] Zhong R., Wan X., Wang D., Zhao C., Liu D., Gao L., Wang M., Wu C., Nabavid S.M., Daglia M. (2020). Polysaccharides from Marine Enteromorpha: Structure and Function. Trends Food Sci. Technol..

[31] Colombo R., Moretto G., Pellicorio V., Papetti A. (2024). Globe Artichoke (*Cynara scolymus* L.) by-Products in Food Applications: Functional and Biological Properties. Foods.

[32] Fang T., Zhang X., Hu S., Yu Y., Sun X., Xu N. (2021). Enzymatic Degradation of *Gracilariopsis lemaneiformis* Polysaccharide and the Antioxidant Activity of Its Degradation Products. Mar. Drugs.

[33] Wu Q., Qin D., Cao H., Bai Y. (2020). Enzymatic Hydrolysis of Polysaccharide from *Auricularia auricula* and Characterization of the Degradation Product. Int. J. Biol. Macromol..

[34] Li H., He W., Xu S., Wang R., Ge S., Xu H., Shan Y., Ding S. (2024). Grafting Chlorogenic Acid Enhanced the Antioxidant Activity of Curdlan Oligosaccharides and Modulated Gut Microbiota. Food Chem. X.

[35] Gao H., Wen J.-J., Hu J.-L., Nie Q.-X., Chen H.-H., Xiong T., Nie S.-P., Xie M.-Y. (2018). Polysaccharide from Fermented *Momordica charantia* L. with *Lactobacillus plantarum* NCU116 Ameliorates Type 2 Diabetes in Rats. Carbohydr. Polym..

[36] Dou Z., Chen C., Fu X. (2019). Bioaccessibility, Antioxidant Activity and Modulation Effect on Gut Microbiota of Bioactive Compounds from *Moringa oleifera* Lam. Leaves during Digestion and Fermentation in Vitro. Food Funct..

[37] Walker A.W., Duncan S.H., McWilliam Leitch E.C., Child M.W., Flint H.J. (2005). pH and Peptide Supply Can Radically Alter Bacterial Populations and Short-Chain Fatty Acid Ratios within Microbial Communities from the Human Colon. Appl. Environ. Microbiol..

[38] Sui Y.-H. (2016). Dietary Saturated Fatty Acid and Polyunsaturated Fatty Acid Oppositely Affect Hepatic NOD-like Receptor Protein 3 Inflammasome through Regulating Nuclear Factor-Kappa B Activation. WJG.

[39] Valguarnera E., Scott N.E., Azimzadeh P., Feldman M.F. (2018). Surface Exposure and Packing of Lipoproteins into Outer Membrane Vesicles Are Coupled Processes in *Bacteroides*. mSphere.

[40] Zafar H., Saier M.H. (2021). Gut *Bacteroides* Species in Health and Disease. Gut Microbes.

[41] Tan H., Zhao J., Zhang H., Zhai Q., Chen W. (2019). Novel Strains of *Bacteroides fragilis* and *Bacteroides ovatus* Alleviate the LPS-Induced Inflammation in Mice. Appl. Microbiol. Biotechnol..

[42] Sun Y., Zhang S., Nie Q., He H., Tan H., Geng F., Ji H., Hu J., Nie S. (2023). Gut Firmicutes: Relationship with Dietary Fiber and Role in Host Homeostasis. Crit. Rev. Food Sci. Nutr..

[43] Holscher H.D. (2017). Dietary Fiber and Prebiotics and the Gastrointestinal Microbiota. Gut Microbes.

[44] Tsai H.-J., Tsai W.-C., Hung W.-C., Hung W.-W., Chang C.-C., Dai C.-Y., Tsai Y.-C. (2021). Gut Microbiota and Subclinical Cardiovascular Disease in Patients with Type 2 Diabetes Mellitus. Nutrients.

[45] Enache R.-M., Profir M., Roşu O.A., Creţoiu S.M., Gaspar B.S. (2024). The Role of Gut Microbiota in the Onset and Progression of Obesity and Associated Comorbidities. Int. J. Mol. Sci..

[46] Nagao-Kitamoto H., Leslie J.L., Kitamoto S., Jin C., Thomsson K.A., Gillilland M.G., Kuffa P., Goto Y., Jenq R.R., Ishii C. (2020). Interleukin-22-Mediated Host Glycosylation Prevents *Clostridioides difficile* Infection by Modulating the Metabolic Activity of the Gut Microbiota. Nat. Med..

[47] Martinon F. (2008). Detection of Immune Danger Signals by NALP3. J. Leukoc. Biol..

[48] Yang X., Zhang M., Liu Y., Wei F., Li X., Feng Y., Jin X., Liu D., Guo Y., Hu Y. (2023). Inulin-Enriched *Megamonas funiformis* Ameliorates Metabolic Dysfunction-Associated Fatty Liver Disease by Producing Propionic Acid. npj Biofilms Microbiomes.

[49] Yan J., Pan Y., He J., Pang X., Shao W., Wang C., Wang R., He Y., Zhang M., Ye J. (2023). Toxic Vascular Effects of Polystyrene Microplastic Exposure. Sci. Total Environ..

[50] Sun W.-M., Wang Y.-P., Duan Y.-Q., Shang H.-X., Cheng W.-D. (2014). *Radix hedysari* Polysaccharide Suppresses Lipid Metabolism Dysfunction in a Rat Model of Non-Alcoholic Fatty Liver Disease via Adenosine Monophosphate-Activated Protein Kinase Pathway Activation. Mol. Med. Rep..

[51] Strowig T., Henao-Mejia J., Elinav E., Flavell R. (2012). Inflammasomes in Health and Disease. Nature.

[52] Teferra T.F. (2021). Possible Actions of Inulin as Prebiotic Polysaccharide: A Review. Food Front..

[53] Verhaegh R., Becker K.A., Edwards M.J., Gulbins E. (2020). Sphingosine Kills Bacteria by Binding to Cardiolipin. J. Biol. Chem..

[54] Madeo F., Hofer S.J., Pendl T., Bauer M.A., Eisenberg T., Carmona-Gutierrez D., Kroemer G. (2020). Nutritional Aspects of Spermidine. Annu. Rev. Nutr..

