# Supplementary material for: Impact of Enzymatic Degradation Treatment on Physicochemical Properties, Antioxidant Capacity, and Prebiotic Activity of Lilium Polysaccharides
Source: Foods. 2025 Jan 14;14(2):246. doi: 10.3390/foods14020246 (PMC11765260; doi:10.3390/foods14020246)
Supplement: Supplementary file 1 [file foods-14-00246-s001.zip › foods-3415705-supplementary.pdf]

Supplementary Materials

Impact of Enzymatic Degradation Treatment on Physicochemical Properties, Antioxidant Capacity, and Prebiotic Activity of Lilium Polysaccharides

Kaitao Peng [1], Yujie Zhang [1], Qi Zhang [1], Yunpu Wang [1], Yuhuan Liu [2,*] and Xian Cui [1,*]

[1] State Key Laboratory of Food Science and Resources, Engineering Research Center for Biomass Conversion, Ministry of Education, Nanchang University, Nanchang 330047, China; pkt1811826@163.com (K.P.); jialishena@163.com (Y.Z.); zhangqi093115@ncu.edu.cn (Q.Z.); wangyunpu@ncu.edu.cn (Y.W.)

[2] Chongqing Research Institute, Nanchang University, Chongqing 402660, China

* Correspondence: liuyuhuan@ncu.edu.cn (Y.L.); cuixian@ncu.edu.cn (X.C.)

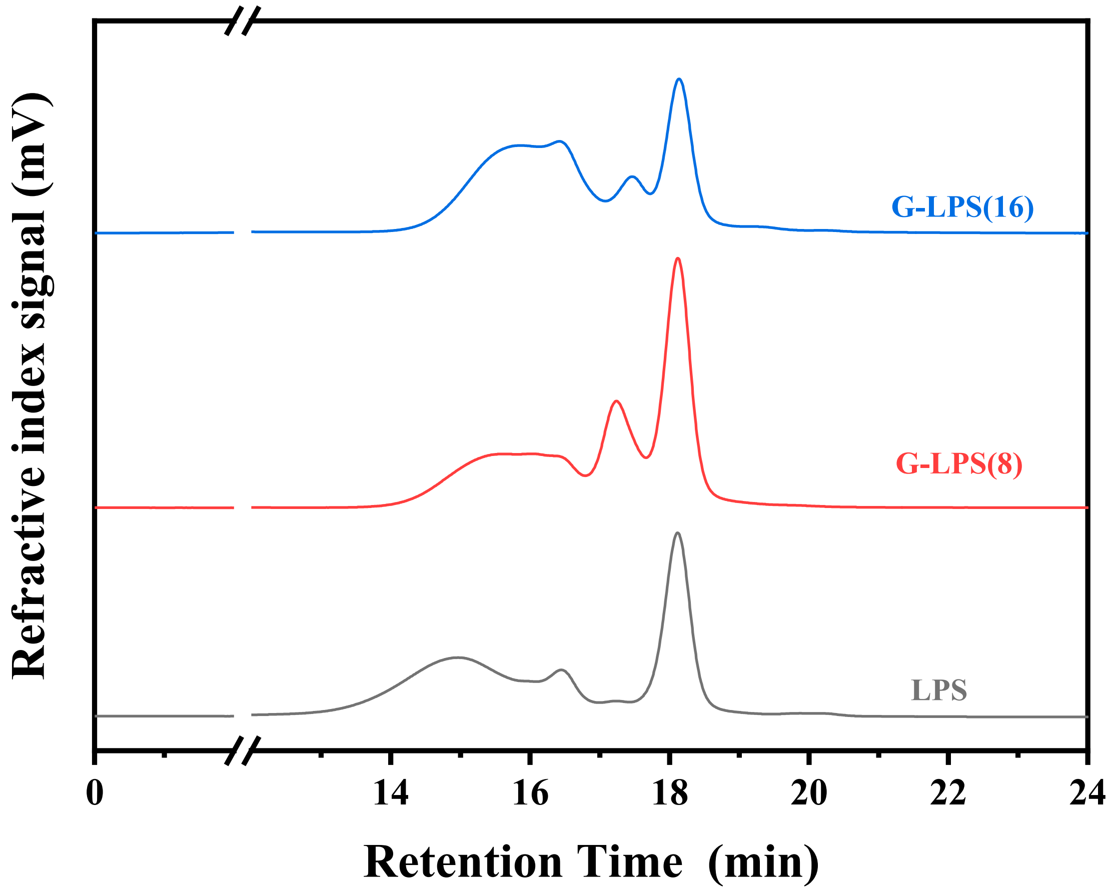

Figure S1. HPGPC elution chromatogram of LPS, G-LPS(8) and G-LPS(16). LPS represents the original *Lilium* polysaccharide, and G-LPS(8) and G-LPS(16) are degradation products of LPS treated with 0.8× 10^3 U/g and 1.6× 10^3 U/g β-glucanase, respectively.

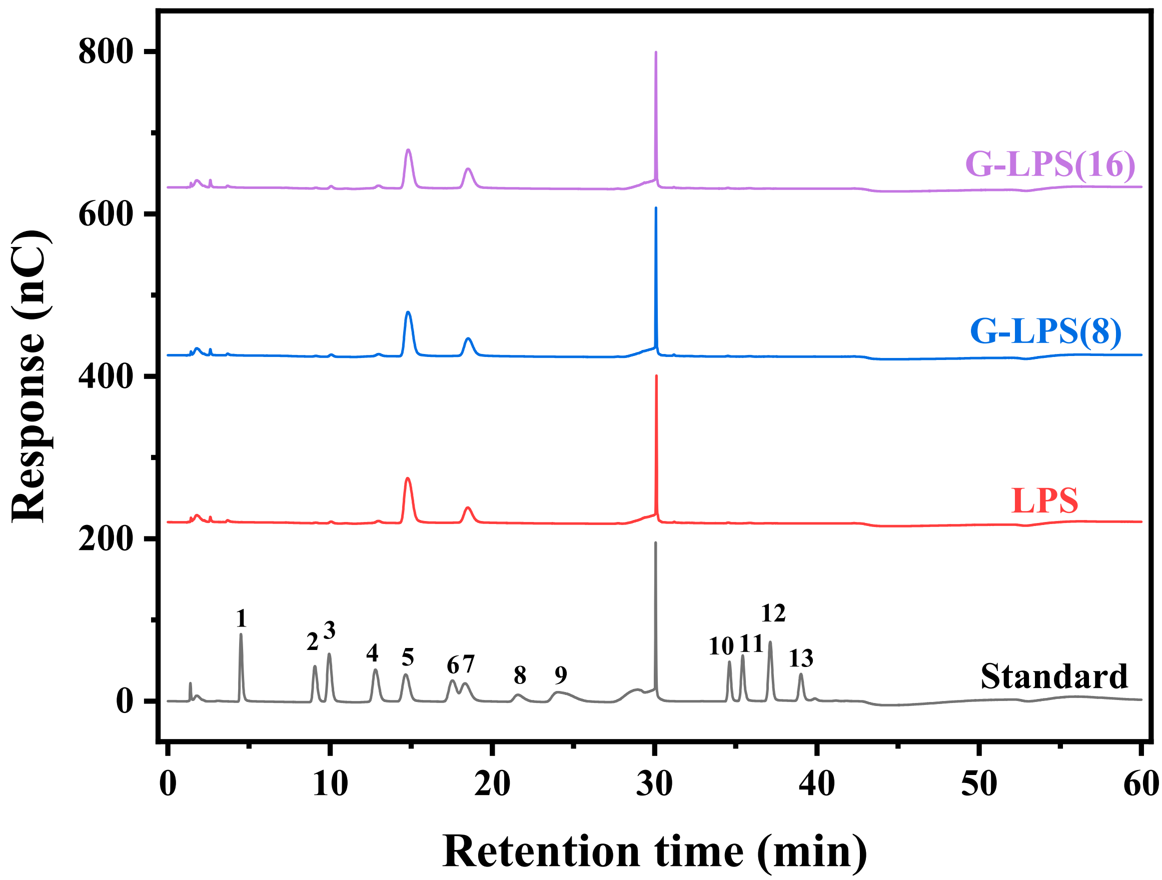

Figure S2. HPAEC chromatograms of standard monosaccharides and LPS, G-LPS(8), G-LPS(16). LPS represents the original *Lilium* polysaccharide, and G-LPS(8) and G-LPS(16) are degradation products of LPS treated with 0.8× 10^3 U/g and 1.6× 10^3 U/g β-glucanase, respectively. The peaks of 1–8 were standards: (1) Fuc, (2) Rha, (3) Ara, (4) Gal, (5) Glc, (6) Xyl, (7) Man, (8) Fru, (9) Rib, (10) Gal-UA, (11) Gul-UA, (12) Glc-UA, (13) Man-UA.

Table S1. Differential metabolites in VIP cluster heatmaps (VIP ≥2, p < 0.05, with FC ≥1).

ID	Metabolite	Superclass	Metab ID	VIP_Plsda	P_value
neg_4179	Zanamivir	Organic acids and derivatives	metab_24822	2.8119	8.62E-05
neg_14831	Oxoglutaric Acid	Organic acids and derivatives	metab_35474	2.552	0.000196
neg_1796	M8-Nelfinavir	Organic acids and derivatives	metab_22439	2.4516	0.000149
neg_14705	Malonic Acid	Organic acids and derivatives	metab_35348	2.3758	7.35E-09
neg_780	3-Oxoglutaric Acid	Organic acids and derivatives	metab_21423	2.239	2.00E-05
neg_13341	Heptenophos	Organic acids and derivatives	metab_33984	2.222	2.37E-06
pos_9855	N-(2-Hydroxyethyl)Eicosa-5,8,11,14-Tetraenamide	Organic acids and derivatives	metab_9854	2.206	0.02142
pos_1830	2-Amino-3-Oxoadipate	Organic acids and derivatives	metab_1829	2.1943	0.000116
neg_767	Hboa Glucuronide	Organic acids and derivatives	metab_21410	2.1714	1.50E-05
neg_13915	3-Methoxyphenol Sulfate	Organic acids and derivatives	metab_34558	2.0996	0.001355
pos_24	Hydroxy Ritonavir	Organic acids and derivatives	metab_23	2.0239	5.41E-06
pos_6061	6-Methylhept-5-En-2-Ol	Lipids and lipid-like molecules	metab_6060	2.2821	3.53E-06
pos_6367	(23S)-23,25-Dihdroxy-24-Oxovitamine D3 23-(Beta-Glucuronide)	Lipids and lipid-like molecules	metab_6366	2.2693	0.04306
pos_1022	Delta-Guanidinovaleric Acid	Lipids and lipid-like molecules	metab_1021	2.2594	1.90E-06
pos_10973	Ganodermic Acid Jb	Lipids and lipid-like molecules	metab_10972	2.2519	0.01161
pos_7059	Curdione	Lipids and lipid-like molecules	metab_7058	2.2306	0.01636
neg_11423	Anthroylouabain	Lipids and lipid-like molecules	metab_32066	2.1415	0.002133
pos_2278	Panthenol	Organic oxygen compounds	metab_2277	2.9885	2.11E-08
neg_651	2-Hydroxy-1,4-Benzoxazin-3-One Glucuronide	Organic oxygen compounds	metab_21294	2.8403	1.89E-05
pos_13997	Ciramadol	Organic oxygen compounds	metab_13996	2.4452	0.004068
neg_14682	6-(3-Carboxy-4-Hydroxyphenoxy)-3,4,5-Trihydroxyoxane-2-Carboxylic Acid	Organic oxygen compounds	metab_35325	2.1691	6.94E-07
pos_14432	Beta-D-Glucopyranuronic Acid	Organic oxygen compounds	metab_14431	2.1652	0.000562

neg_13146	Shikimic Acid	Organic oxygen compounds	metab_33789	2.1571	1.95E-05
pos_573	Glycogen	Organic oxygen compounds	metab_572	2.0908	4.81E-07
pos_7144	4-Methylumbelliferone	Phenylpropanoids and polyketides	metab_7143	2.4614	0.1046
pos_6871	31-O-Demethyltacrolimus	Phenylpropanoids and polyketides	metab_6870	2.3464	3.90E-05
neg_3351	7-Hydroxy-5-Methoxy-4-Methyl-3-(4-Methylpiperazin-1-Yl)-2H-Chromen-2-One	Phenylpropanoids and polyketides	metab_23994	2.1141	0.000172
pos_8766	Formononetin	Phenylpropanoids and polyketides	metab_8765	2.0922	2.85E-05
neg_11067	Edulisin Vi	Phenylpropanoids and polyketides	metab_31710	2.0259	0.000249
pos_7379	13-Demethyl Tacrolimus	Organoheterocyclic compounds	metab_7378	2.7102	4.25E-08
neg_13895	Buprenorphine Glucuronide	Organoheterocyclic compounds	metab_34538	2.5018	0.000277
pos_2936	P-Hydroxyl-Ethotoin	Organoheterocyclic compounds	metab_2935	2.3508	7.96E-08
neg_2270	2-Furoic Acid	Organoheterocyclic compounds	metab_22913	2.1648	0.000511
pos_7638	4-Methylcatechol	Benzenoids	metab_7637	2.678	6.20E-05
pos_6908	Pioglitazone	Benzenoids	metab_6907	2.276	0.01164
neg_4514	6-[Ethoxy(Mercapto)Methylidene]-1-Cyclohexa-2,4-Dienone	Benzenoids	metab_25157	2.1997	0.001827
pos_8682	1-Isothiocyanato-6-(Methylsulfinyl)Hexane	Others	metab_8681	2.4337	0.004721
pos_487	Agmatine	Others	metab_486	2.404	0.000587
neg_10427	3'-Deoxy-3'-Fluorothymidine	Others	metab_31070	2.2482	4.42E-07
pos_10612	N-Linoleoyl Lysine	Others	metab_10611	2.1944	0.009899
pos_10922	Cerp(D17:0/2:0)	Others	metab_10921	2.1496	0.000976
pos_19231	Phe Met Gly	Others	metab_19230	2.0666	3.29E-05
pos_19506	Glu Thr Ala	Others	metab_19505	2.0597	0.000463
neg_10978	1,1-Dichloroheptane	Others	metab_31621	2.6996	3.81E-05
